# Dietary Antioxidants, Macular Pigment, and Glaucomatous Neurodegeneration: A Review of the Evidence

**DOI:** 10.3390/nu11051002

**Published:** 2019-05-01

**Authors:** Thomas Lawler, Yao Liu, Krista Christensen, Thasarat S. Vajaranant, Julie Mares

**Affiliations:** 1 Department of Nutritional Sciences, University of Wisconsin, 1415 Linden Drive, Madison, WI 53706, USA; tlawler2@wisc.edu; 2 Department of Ophthalmology and Visual Sciences, University of Wisconsin, 2870 University Avenue, Madison, WI 53705, USA; yao.liu2@wisc.edu; 3 Department of Ophthalmology and Visual Sciences, University of Wisconsin, 610 N. Walnut Street, 1069 WARF Building, Madison, WI 53726, USA; krista.christensen@wisc.edu; 4 Department of Ophthalmology and Visual Sciences, University of Illinois – Chicago, 1855 W. Taylor Street, Chicago, IL 60612, USA; thasarat@uic.edu

**Keywords:** glaucoma, antioxidants, oxidative stress, macular pigment, lutein

## Abstract

Primary open-angle glaucoma (POAG) is a leading cause of irreversible blindness worldwide, and the prevalence is projected to increase to 112 million worldwide by 2040. Intraocular pressure is currently the only proven modifiable risk factor to treat POAG, but recent evidence suggests a link between antioxidant levels and risk for prevalent glaucoma. Studies have found that antioxidant levels are lower in the serum and aqueous humor of glaucoma patients. In this review, we provide a brief overview of the evidence linking oxidative stress to glaucomatous pathology, followed by an in-depth discussion of epidemiological studies and clinical trials of antioxidant consumption and glaucomatous visual field loss. Lastly, we highlight a possible role for antioxidant carotenoids lutein and zeaxanthin, which accumulate in the retina to form macular pigment, as evidence has emerged supporting an association between macular pigment levels and age-related eye disease, including glaucoma. We conclude that the evidence base is inconsistent in showing causal links between dietary antioxidants and glaucoma risk, and that prospective studies are needed to further investigate the possible relationship between macular pigment levels and glaucoma risk specifically.

## 1. Introduction

Glaucoma is a progressive neurodegenerative disease in which the death of retinal ganglion cells (RGCs) in the inner retina results in characteristic visual field defects that can progress to blindness [1]. It is a leading cause of irreversible blindness worldwide and the incidence of glaucoma is expected to increase as the population ages, with a projected prevalence of 112 million worldwide by 2040 [2]. Healthcare spending related to glaucoma is estimated at roughly $5.8 billion per year in the U.S. alone [3]. Moreover, vision loss from glaucoma may make it difficult to perform activities of daily living, limit independence, and increase the risk of falls [4], depression [5], and cognitive decline [6]. Due to the lack of symptoms in the early stages, detection and treatment may be delayed until advanced stages of disease [7]. It is estimated that half of patients with glaucoma are unaware of their diagnosis [8].

Nonmodifiable glaucoma risk factors include older age, race/ethnicity (e.g., African, Asian, or Hispanic heritage), family history of glaucoma, and central corneal thickness [9]. Current treatments for glaucoma are limited to lowering of intraocular pressure (IOP) using medications, laser treatment, or surgery [7], but these interventions are not always sufficient. Some patients continue to experience disease progression and severe vision loss can occur despite achieving therapeutic IOP lowering. The identification of novel modifiable risk factors has significant implications for glaucoma prevention and may lead to new treatment modalities. In recent years, new evidence has emerged of an association between markers of oxidative stress and risk for primary open-angle glaucoma (POAG) [10,11], suggesting that consumption of dietary antioxidants may be a factor capable of modifying disease risk.

We review the growing body of evidence linking oxidative stress to the pathophysiology of POAG and review human studies linking dietary intake of antioxidants to risk for POAG. In later sections, we focus specifically on dietary carotenoids lutein and zeaxanthin (L/Z), given their well-established antioxidant activities in the retina and the ability to noninvasively estimate the density of these carotenoids within the macular region of the retina (termed macular pigment (MP)), where they accumulate at the highest density. More recent cross-sectional studies have linked MP to a reduced risk for prevalent POAG, suggesting that dietary modifications to increase MP may be one approach to lower glaucoma risk in individuals with elevated IOP [12,13].

## 2. Pathophysiology of Glaucoma

POAG is the most common form of glaucoma in the U.S. and worldwide [1]. In POAG, the iridocorneal angle where drainage of the aqueous humor occurs remains visibly open, yet IOP may be elevated due to either excess production of aqueous humor by the ciliary body or an impediment of aqueous humor outflow through the trabecular meshwork into the canal of Schlemm [1]. The optic nerve head, where RGC axons pass through the porous connective tissue plates of the lamina cribrosa and leave the eye to form the optic nerve in the brain, is likely the initial site of IOP-related RGC injury [14]. Elevated IOP may interfere with retrograde transport of neurotropic factors, such as brain derived neurotrophic factor [15,16]; alter cytoskeletal structure [16]; and/or induce mitochondrial dysfunction [17]. These consequences of elevated IOP can induce a bioenergetic crisis, disturb normal axonal transport, and activate RGC cell death pathways.

Although IOP reduction through the use of topical medications and/or surgery is effective, many individuals continue to develop glaucoma despite having normal eye pressures and incur progressive glaucomatous vision loss despite treatment, suggesting that there are other mechanisms contributing to RGC death. These other biological insults may include chronic inflammation, hypoxia, excitotoxicity, loss of neurotrophic factors, and oxidative stress [18] that may be independent of pressure related injury. Consequently, there may be opportunities to develop novel treatment approaches to use in conjunction with pharmaceutical or surgical approaches to reduce IOP. A large body of evidence implicates oxidative stress, as there is evidence for oxidative damage (and depletion of antioxidants) in the aqueous humor [19,20] and trabecular meshwork [21,22] of glaucoma cases compared with age and sex-matched controls.

## 3. Oxidative Stress in Primary Open-Angle Glaucoma Pathogenesis

Current evidence suggests that oxidative stress is relevant to the pathophysiology of POAG and may contribute to damage in the retina and the anterior chamber of the eye (the space between the iris and the corneal endothelium containing aqueous humor). An imbalance between reactive oxygen species (ROS) and antioxidants in these tissues can result in excessive generation of ROS, including hydrogen peroxide, superoxide, and peroxynitrate, tipping the balance in favor of oxidative stress. These insults may damage cellular macromolecules (nucleic acids, structural proteins, and lipids [23]) and organelles, including mitochondria, and may promote cell death via apoptosis [24]. Prior studies have observed diminished total antioxidant capacity in plasma or serum of glaucoma cases compared to matched controls [10,11,25,26], as well as increased levels of oxidation products in the serum (e.g., malonyldialdehyde, a lipid peroxidation product) [25,26,27]. The total antioxidant capacity in aqueous humor has been observed to be lower in POAG patients compared to age- and sex-matched controls [20,25,26], along with levels of some specific antioxidants including vitamins C and E [28]. There is evidence for greater activity of antioxidant enzymes (glutathione peroxidase and superoxide dismutase) in the aqueous humor of glaucoma cases compared to control individuals [19,20,28], which may signal the body’s attempt to combat oxidative stress [20]. In a recent meta-analysis containing pooled data from 22 case–control studies, there was consistent evidence for a significant increase in oxidative stress markers (e.g., malonyldialdehyde) and a decrease in antioxidant level in both serum and aqueous humor in cases from several glaucoma subtypes (including but not limited to POAG) compared to age- and sex-matched controls [29].

Mechanisms by which oxidative stress may contribute to glaucomatous neurodegeneration are complex and multifactorial. One mechanism by which oxidative stress may contribute to glaucoma is by damaging cells in the trabecular meshwork [30], leading to interference with normal drainage of aqueous humor [31] and an increase in IOP [21]. Consistent with oxidative stress in the aqueous humor, there is evidence that cells within the trabecular meshwork of glaucoma cases have higher levels of 8-hydroxy-2′-deoxyguanosine (8-OHdG) [21,22], a marker of oxidative damage to the DNA. Oxidative damage to trabecular meshwork cells may cause remodeling of the cytoskeleton [30] and decrease outflow facility [31] and it has been observed that presence of 8-OHdG in trabecular meshwork cells correlates with several measures of glaucoma disease severity, including degree of visual field loss and IOP [21,22].

Oxidative stress may also contribute directly to the apoptotic death of RGCs of the inner retina [32] and activation of the immune system [33,34,35]. Animal models of glaucoma suggest that elevated IOP causes oxidative stress in RGCs [32,36,37]. In a rat model of elevated IOP, induced by injection of hyaluronic acid in the anterior chamber, retinal levels of antioxidant enzymes SOD and catalase were decreased over 10 weeks, as were levels of glutathione, while markers of lipid peroxidation were markedly increased [36]. Another group reported that elevated IOP (induced in rats by episcleral vein cauterization) can increase levels of malonyldialdehyde and the free radical superoxide [32], while others have reported that acute elevation of IOP or hydrostatic pressure can induce markers of oxidative stress in both mouse retina and RGC-5 cell culture in a matter of hours [38].

Unstable perfusion of the optic nerve head and retina, characterized by large fluctuations in the ocular perfusion pressure, has emerged as another source of oxidative stress that may cause glaucomatous damage, evidenced by new imaging technologies that allow measurement of retinal vessel caliper, ocular blood flow, and ocular perfusion pressure [39]. Vascular diseases, including stroke, cardiovascular disease, migraine, and hypertension, are associated with increased risk for glaucoma, strongly suggesting that vascular abnormalities and impaired blood flow to the retina play a role in development of the disease [39]. Decreased ocular blood flow is a risk factor for prevalent glaucoma in large epidemiological studies [40,41], as well as progression of glaucoma over time [42]. Similar associations are seen for vessel narrowing [43]. It has also been noted that glaucoma is associated with primary vascular dysregulation [44,45], leading to speculation that unstable perfusion of the retina may be an important mechanism in damage to the optic nerve head. Unstable retinal blood flow may lead to oxidative stress-related injury, as periods of relative ischemia may be succeeded by periods of reperfusion in which blood supply to the retina exceeds the need for oxygen, contributing to the formation of ROS [46].

Mitochondrial dysfunction in RGCs may also contribute to the generation of ROS [47], as mitochondria are abundant in RGC axons to provide ATP for sodium/potassium ATPase activity needed to maintain electrical conductivity, and to support axonal transport of organelles and other materials [48]. Mitochondria are the cell’s primary consumers of oxygen, and hence contribute significantly to generation of reactive oxygen species. Glaucoma patients have significantly lower rates of complex-1 driven ATP synthesis, which is inversely associated with disease severity [49]. Further, glaucoma cases may have higher rates of mitochondrial DNA (mtDNA) mutations and lower mean mitochondrial respiratory activity than controls [50], consistent with mitochondrial dysfunction and oxidative stress.

Oxidative stress may promote RGC death directly by activating apoptotic cell signaling pathways [51], or indirectly by interacting with the retinal immune system [33,34,35,52]. Oxidative stress arising from glaucomatous stimuli can cause retinal glial cells (including Mueller cells and astrocytes) to adapt an ‘immune-activated’ phenotype, characterized by greater expression of major histocompatibility complex (MHC) II [52] and toll-like receptors [33]. In cell culture experiments, oxidative stress increases the expression of MHCII by glial cells, consequently improving their ability to stimulate T cell proliferation and secretion of cytokines (including TNF-α) that can induce ganglion cell apoptosis [52]. Activation of the innate and adaptive immune systems under conditions of cell stress may help to promote homeostasis and the removal of dead or dying cells. However, it has also been suggested that chronic immune activation under pathologic conditions may also exacerbate the disease state in glaucoma [34].

## 4. Dietary Antioxidants and Risk for POAG

Given the body of evidence linking oxidative stress to the etiology of POAG, several researchers have proposed the potential utility of antioxidant treatments [53,54]. However, there is limited evidence that long-term antioxidant intake lowers risk for glaucoma incidence or severity. Four large-scale epidemiological studies have examined this relationship using estimated dietary intake and a handful of intervention trials have been conducted (see Table 1).

### 4.1. Epidemiological Studies

A cross-sectional analysis of data from the National Health and Nutrition Examination Survey showed a statistically significant decrease in the odds of self-reported glaucoma among those currently using vitamin C supplements, with no association seen for use of vitamin A or E supplements, and no association for levels of vitamins A, C, or E in serum; carotenoid use in supplements or serum levels of carotenoids were not assessed in this study [56]. These analyses were limited by reliance on self-reported glaucoma as the main outcome measure, which is known to be of limited accuracy and many individuals with glaucoma are unaware of the disease, limiting the ability to detect an association if it exists [8]. Also, given that glaucoma may take decades to develop, it is unlikely that the use of antioxidant containing supplements at any single point in time would be an etiologically relevant exposure.

In the Nurses’ Health Study (NHS) and Health Professionals Follow-up Study (HPFS), which included 116,484 participants ≥40 years of age, there was no strong evidence that dietary intake of carotenoids (α-carotene, β-carotene, β-cryptoxanthin, lycopene, and L/Z) or antioxidant vitamins (A, C, and E) was associated with risk for incident glaucoma over an average follow-up period of 9.1 years, except for vitamin E from foods (odds ratio (OR) of 0.67 comparing highest to lowest quintile of intake) [55]. The study authors made a considerable effort to prevent misclassification, by excluding individuals who did not attend regular eye checkups, and by confirming cases of self-reported glaucoma by reviewing medical records (including visual fields). Although this should be considered a strength of this study, it is still possible that misclassification of participants (especially those unaware that they had glaucoma) may have biased the results towards the null. It is also not clear that results from a cohort of healthcare professionals generalize to other populations due to differences in diet and other health-related behaviors; perhaps most importantly, dietary intake of antioxidants may not reflect the quantity available to the eye due to differences in absorption and transport of antioxidants related to genetic heterogeneity [62,63], bioavailability [64,65], and reliance on circulating lipoproteins for transport of lipid soluble molecules (e.g., carotenoids) [66,67,68]. In this study, it was not determined whether serum antioxidant levels were predictive of incident glaucoma.

In the Rotterdam Study, glaucoma was assessed by visual field testing and 91 of 3502 eligible participants, age 55 years and older, developed glaucoma over an average of 9.7 years of follow-up [59]. There was no association between the intake of the majority of antioxidant carotenoids and vitamins evaluated (including carotenoids α-carotene, β-carotene, lutein, zeaxanthin, and β cryptoxanthin, and vitamins C and E) and risk for glaucoma, although protective associations were observed for thiamine and retinol equivalents. This study is limited by lack of serum antioxidant measures, and may have been underpowered given the relatively small number of glaucoma cases. 

A cross-sectional analysis of African American women greater than 65 years old participating in the Study of Osteoporotic Fractures found that specific antioxidant-rich fruits and vegetables (including oranges, peaches, and collard greens/kale) were associated with reduced risk for prevalent glaucoma (*p* < 0.05), ascertained via visual field testing and the appearance of the optic nerve head [61]. Specific dietary antioxidant vitamins and carotenoids (vitamins A and C and α-carotene) were also associated with significantly reduced risk after adjustment for potential confounders. Trends were also observed for β-carotene and L/Z, although the trends did not reach significance (*p* = 0.05–0.08). In an earlier report from the Study of Osteoporotic Fractures, including 1155 participants of Caucasian or African American background, there was a significant protective association for collard greens/kale (OR: 0.31 for ≥1 serving per month) [60]. α-carotene and β-carotene were also inversely associated with glaucoma risk in this sample, although the association between L/Z consumption and risk for glaucoma did not approach significance in this sample.

In summary, there is limited evidence from epidemiological studies that consumption of dietary antioxidants is associated with reduced risk for incident or prevalent glaucoma. Inconsistencies may arise from differences in diagnostic criteria to ascertain glaucoma cases (i.e., visual field testing vs. self-report) and heterogeneity in the racial/genetic background of participants, which may influence genetic disease risks and dietary habits. Measuring diet during the lengthy, etiologically relevant time frame for glaucoma remains a persistent challenge in nutritional epidemiology, and this may be considered a limitation in cross-sectional studies of glaucoma prevalence [56,60,61].

### 4.2. Clinical Trials

There have been few randomized controlled trials (RCTs) to determine whether the administration of high dose antioxidant supplements can prevent new cases of glaucoma or slow the progression of visual field loss in glaucoma patients. In one trial, a supplement containing B-vitamins; vitamins A, C, and E; carotenoids; and antioxidant minerals with or without omega 3 fatty acids consumed over 2 years, did not prevent visual field loss or thinning of the retinal nerve fiber layer or ganglion cell complex (GCC) in a study of older adults with glaucoma [57]. There is more evidence from trials of Ginkgo Biloba extract (GBE) indicating that this substance may be a useful therapeutic tool for those with glaucoma, possibly slowing the rate of visual field loss and thinning of the neuronal retina [69,70,71,72,73,74]. Results from animal studies are also supportive, as the administration of GBE can prevent RGC death related to chronic elevation of IOP and destruction of the optic nerve [70,72]. GBE has approximately 60 bioactive substances, including antioxidants, and may possibly influence glaucoma progression through several unique mechanisms, including effects on ocular blood flow [73].

## 5. Antioxidant Effects of Lutein and Zeaxanthin in the Retina

Although research consistently demonstrates increased oxidative stress and depletion of antioxidants in the serum and aqueous humor of adults with glaucoma [29], there is not consistent evidence from epidemiological or clinical studies to implicate specific antioxidants. However, a growing body of evidence from animal models and cell culture indicates that antioxidant carotenoids L/Z, which uniquely accumulate in the retina in humans, may prevent RGC death related to glaucomatous stimuli [75,76,77,78,79].

L/Z are polar dietary carotenoids and potent antioxidants that may hold special relevance for retinal health, integrity, and long-term risk for age-related eye disease [80]. L/Z are unique among other major carotenoids found in serum in that they cross the blood–retinal barrier and bind to specific binding proteins in the retina [81,82]. Their highest concentration in human body is located in the macular region located centrally in the retina where they contribute to the pigmentation level (hence the term macular pigment), but macular concentrations of these carotenoids are also correlated with levels in peripheral retinal areas [83]. MP peaks in the center fovea and rapidly declines toward the periphery, and is generally undetectable at 7 degrees from the foveal center [84]. Although the majority of MP is concentrated in the outer retina, in the photoreceptor outer segments and outer plexiform layer, considerable amounts of MP are detectable in the inner plexiform layer [85], adjacent to the ganglion cell layer that is damaged in glaucoma. Due to a backbone of conjugated carbon–carbon double bonds, L/Z act as potent retinal antioxidants [86], able to scavenge ROS including superoxide, hydroxyl radical, and hydrogen peroxide without being ‘used up’ in the process [80]. L/Z also have the unique capacity to filter short-wavelength blue light before it reaches the outer retina [87], thought to protect the retinal pigment epithelium and photoreceptors from the effects of photooxidative stress [88,89]. MP is modifiable with diet and supplement interventions [90,91], and has become an attractive target to treat or prevent age-related eye diseases that have high levels of oxidative stress.

## 6. Lutein and Zeaxanthin and POAG

### 6.1. Protective Mechanisms of L/Z in the Retina

Evidence, which links these carotenoids to diseases of the inner retina, like glaucoma, is now emerging. A recent study of Chinese adults demonstrated that serum concentrations of L/Z, but not other carotenoids, were positively associated with measures of retinal blood flow [92]. This provides preliminary evidence that L/Z may help to stabilize retinal perfusion, which has been linked to glaucoma risk [45]. Additional evidence has emerged that L/Z may also have protective effects on the inner retina in response to glaucomatous stimuli in animal models [75,76,79], with additional evidence that the mechanism of protection involves suppression of apoptotic signaling pathways. Lutein reduced the number of TUNEL-positive RGCs (a marker of apoptotic cell death) in mouse retina after ischemia/reperfusion injury (2 h of retinal ischemia followed by 22 h of reperfusion) and increased the number of viable cells to approximately control levels [75]. These results corroborate those of an earlier study in rats, in which intravitreous or intraperitoneal lutein almost completely prevented RGC loss after 1 h of ischemia and 24 h of reperfusion [93]. Likewise, lutein significantly increased the number of viable RGCs after intravitreal injection of an NMDA receptor agonist, meant to stimulate excitotoxic cell death in the inner retina, although cell viability did not approach control levels [79]. Reduced expression of the proapoptotic Bax protein and increased expression of antiapoptotic Bcl2 in the inner retina mirrored this finding, as well as reduced release of proapoptotic cytochrome C from the mitochondria, a key player in the activation of caspases, the primary effectors of apoptotic cell death. Similar effects on Bax and Bcl2 expression were seen in a cell culture model of rat Mueller cells (a prominent glial cell type in the retina whose activity is linked to onset of glaucomatous pathology), where it was also observed that lutein could reduce levels of cleaved caspase 3 after simulated hypoxia [94]. Antiapoptotic effects of lutein were also apparent in a model of retinal detachment, as lutein treatment reduced levels of cleaved caspase 3 and 8 [95].

While the results from animal models indicate that administration of L/Z may protect the inner retina from glaucomatous insults, the relevance for humans is unclear. Unlike humans, rodents do not accumulate MP in appreciable quantities (although mouse models of MP have recently been developed) [96], and animal models of RGC death based on short-term elevations in IOP or ischemia do not perfectly mimic the complex mechanisms that underlie human glaucoma. Cell culture models of physiologically relevant L/Z concentrations show that L/Z can maintain murine RGC-5 cell viability after cell stress, including simulated hypoxia and serum deprivation [77,97], helping to corroborate results from animal studies. However, cell culture does not perfectly mimic disease pathophysiology, and RGC-5 cells lack several key characteristics of human RGCs, including excitability [98].

### 6.2. L/Z and POAG—Epidemiological Studies

Several groups have evaluated the association between L/Z consumption and risk for POAG in epidemiological studies, with mixed results. In data pooled from the NHS and HPFS, individuals in the highest quintile of dietary L/Z had lower risk than individuals in the lowest quintile OR = 0.68. CI: 0.49–0.93), after excluding the four most recent years of dietary data (to account for a lag in disease diagnosis) [55]. Although there was no significant linear trend, these results are consistent with a possible threshold effect. In contrast, there was no association between for L/Z and glaucoma in the Rotterdam Study [59]. More recently, greater consumption of green leafy vegetables, the predominant source of L/Z in the diet, was linearly associated with reduced risk for incident POAG (*p*-trend = 0.01) in the NHS and HPFS [58]. However, the authors attribute this beneficial association to the presence of nitrates in leafy green vegetables, hypothesized to influence ocular blood flow and regulation of IOP, rather than L/Z content. There was a significant negative association between dietary nitrate consumption (OR: 0.67 for Q5 vs. Q1) and risk of POAG that maintained statistical significance after adjustment for dietary L/Z and other antioxidants. However, it may be difficult to identify independent effects among highly correlated nutrient intakes, and differences in the degree of measurement error between nutrients exacerbate this difficulty. Other groups have also observed a negative association for leafy green vegetables, and it is not clear whether this is attributable to the L/Z content, nitrates, and/or another bioactive component [60,61].

Accumulation of L/Z in the retina as MP may be a more relevant exposure metric than L/Z in the diet, as the quantity of L/Z that reaches the retina is highly dependent on genetic heterogeneity [99,100] and other factors, including body composition and metabolic health [101,102]. Several low-cost, noninvasive approaches to reliably measure MP have been developed in recent years [103], making it feasible to investigate MP level as a potential modifiable risk factor for POAG. Currently, the literature contains only four studies directly addressing the association between MP and the presence of POAG and glaucomatous optic neuropathy (see Table 2). In the first, a study of 40 glaucoma cases and 54 normal controls, it was determined that MP optical density (MPOD), assessed using customized heterochromatic flicker photometry (cHFP), was approximately 36% lower in cases, although there was no association with disease severity [12]. However, in a follow-up study of 88 glaucoma cases, they discovered that MPOD at multiple eccentricities was significantly lower (*p* < 0.0001) in cases with foveal GCC loss (an indicator of greater disease severity) compared to cases without foveal involvement [13]. Although the associations with disease severity differ in these studies, the authors suggest that the differences in sample size and methodology may explain the different results, as the first study did not include optical coherence tomography scans to assess foveal GCC loss. In the second study, MPOD was also significantly and positively associated with glaucoma-related structural parameters including optic disc rim area, GCC thickness, and thickness of the inner retina, although the results were only marginally significant (*p* = 0.02–0.05). MPOD was negatively associated with cup to disc ratio, suggesting maintenance of RGC number. The results obtained by this group were later corroborated by another group utilizing the one-wavelength reflectometry approach for measuring MP, as both maximum and mean MPOD were reduced in 30 POAG patients relative to 52 controls, by 19% and 14%, respectively [104]. However, in the recent San Diego Macular Pigment Study, MP volume did not differ between 85 glaucoma patients and 22 controls when measured utilizing the two-wavelength autofluorescence technique [105]. There was also no association between MP volume and retinal nerve fiber layer thickness, as assessed by optical coherence tomography imaging of the retina.

It is not clear why there have been inconsistent results, but it is possible that reliance on different testing approaches for MPOD may have led to different conclusions [103]. Some approaches are biased by the health of the lens and retina [103]. While cHFP is reliable in older adults [106] and controls for differences in lens density and retinal health [103], it is time-consuming, possibly leading to participant fatigue, and otherwise relies on the skill of the examiner and patient to produce valid measurements. It is not known whether the peculiar changes to retinal health and vision function that occur in glaucoma could also bias results of MPOD testing, or whether bias would be limited to cases of late-stage, foveal-involved glaucoma, as MP accumulates almost exclusively in the fovea [107]. Given the inconsistent results of MP levels in relation to glaucoma and the small samples used to evaluate this association, this is an interesting area for further investigation.

As all of these are studies cross-sectional, it is not possible to determine whether high MPOD is associated with risk for incident glaucoma over time. To help clarify this issue, long-term prospective studies of MP and glaucomatous visual field loss and changes to the retina are required. In addition, reverse causality cannot be ruled out, as glaucomatous changes to the retina, including thinning of the GCC and impaired blood flow to the retina, may make it difficult to accrue MP. Although MP attenuates oxidative stress by filtering short wavelength light and by directly neutralizing ROS [80], the biological rationale for a protective effect is not entirely clear, as the primary site of RGC injury is believed to be located at the optic nerve head [14], rather, not within the retina. Further, although L/Z accumulate throughout the macula, concentrations are highest in the photoreceptors of the outer retina and in the fovea [107], which usually remains unaffected until later stages of glaucoma [108]. Despite this, multiple studies showing an inverse association between MPOD and risk for glaucoma provide a strong rationale for prospective studies, as interventions to increase MPOD would be a low cost and low risk approach to prevent disease.

## 7. Conclusions and Future Directions

Given the increasing prevalence of glaucoma among older adults and lack of known modifiable risk factors other than lowering of IOP, interventions, such as increasing consumption of antioxidants targeted to those who are at high risk for disease development or progression, may have a considerable population health impact. Despite the evidence supporting oxidative stress in glaucoma, there is still inconsistent evidence as to whether dietary antioxidant intake predicts the incidence or prevalence of POAG, and no prospective evidence that interventions to increase antioxidant consumption can prevent or slow glaucomatous visual field loss or thinning of the neural retina. This may reflect persistent challenges in accurately measuring dietary intake, as well as high levels of interindividual variability in the absorption of antioxidants. Future prospective studies of serum antioxidants and incident visual field loss, or thinning of the inner retina, may help to clarify the effect of antioxidants on the health of the neural retina. Older adults at increased risk for glaucoma are currently advised to consume an overall healthy diet rich in fruits and vegetables, as this diet pattern is linked to reduced risk [61,109], and because maintaining a healthy body weight and good metabolic health through diet and exercise may help to prevent disease.

Despite the limitations of the current evidence, there is a strong rationale for continuing to investigate MP as risk factor for POAG, given its unique antioxidant/protective activities in the retina [80], and ease of modification with diet [91,110] and supplements [90]. The majority of recent cross-sectional research, made possible by low-cost, noninvasive approaches to MPOD testing, suggests an association between higher MPOD and reduced risk for POAG [12,13,104,105]. A protective association is also supported by observations that L/Z may protect the neural retina from glaucomatous stressors, at least in animal models [75,79]. There is currently a need for prospective studies of MPOD and glaucomatous visual field loss to further elucidate whether the relationship is likely to be causal in humans. There is a strong precedent for this type of research, given longstanding interest in MPOD as a modifiable risk factor for AMD (a disease characterized by oxidative stress), and evidence from large-scale clinical trials that high doses of L/Z (12 mg/day) may reduce risk for progression of AMD in some individuals [111] without increased risk for side effects [112]. Prospective studies showing reduced risk for visual field loss with higher MPOD may support the development of low-cost interventions to increase MPOD in individuals at high risk for POAG, administered alongside treatment to lower IOP, and monitoring of MPOD in a clinical setting.

## Figures and Tables

**Table 1:** Summary of human studies of dietary antioxidants (and antioxidant food sources) and primary open-angle glaucoma.

Author, Year	Study Design	Participants (age)	Exposure/Treatment	Outcome	Follow-Up Period	Significant Findings (*p* < 0.05)
Kang, 2003 [55]	Cohort	116,505 health professionals free from glaucoma (≥40 years)	Dietary antioxidant vitamins and carotenoids: Vitamins A, C, E, α and β carotene, lutein/zeaxanthin, lycopene, and β-cryptoxanthin (Total and from foods only)	Incident self-reported primary open-angle glaucoma confirmed by medical record review (*n* = 474)	9.3 years (average)	Vitamin E (foods only) (*p*-trend = 0.02) OR: 0.67 (quintile 5 vs. 1)
Wang, 2013 [56]	Cross-sectional	2912 NHANES participants (≥40 years)	Vitamins A, C, E (from supplements) Vitamins A, C, E (in serum)	Self-reported glaucoma (*n* = 203)	N/A	Vitamin C from supplements (*p* = 0.13) OR: 0.34 (quartile 1 vs. no consumption)
Garcia-Medina, 2015 [57]	RCT	117 POAG cases	Placebo (*n* = 63) Supplement containing vitamins, A, C, E, and B-complex, lutein and zeaxanthin, zinc, copper, selenium, and omega 3 fatty acids (*n* = 54)	-Visual fields: Mean deviation, pattern standard deviation -Retinal nerve fiber layer thickness -Ganglion cell complex thickness	2 years	No effect of treatment was observed for any of the three outcomes.
Kang, 2016 [58]	Cohort	104,987 health professionals (≥40 years)	Total green leafy vegetables	Incident self-reported open-angle glaucoma confirmed by medical record review (*n* = 1483)	16.0 years (average)	Green leafy vegetables inversely associated with glaucoma risk (*p*-trend = 0.02) OR 0.82, (quintile 5 vs. 1)
Ramdas, 2012 [59]	Cohort	3502 adults without POAG (≥55 years)	Dietary antioxidant vitamins and carotenoids: α and β carotene, lutein/zeaxanthin, β-cryptoxanthin, retinol, vitamins B1, B6, B12, C, and E.	Incident POAG, detected by visual field testing (*n* = 91)	9.7 years (average)	Vitamin B1 (*p* = 0.03) HR: 0.31 (tertile 3 vs. 1) Retinol equivalents (*p* = 0.01) HR: 0.33 (tertile 3 vs. 1)
Coleman, 2008 [60]	Cross-sectional	1155 women (67–97 years)	Dietary fruits and vegetables Dietary vitamins and carotenoids (vitamins A, C, E, B-complex, α and β carotene, lutein/zeaxanthin, cryptoxanthin, and lycopene	Prevalent POAG, detected by visual field testing and optic disc examination (*n* = 95)	N/A	Canned/dried peaches (*p* = 0.04) OR: 0.53 (≥1 serving/week, vs. <1/month) Fresh carrots (*p* = 0.009) OR: 0.36, (>2 servings/week vs. <1/month) -Collard greens/kale (*p* = 0.34) OR: 0.31 (≥1 serving/month vs. <1/month) -Vitamin B2 (*p* = 0.02) OR: 0.19 (≥2 mg/day vs. <1 mg/day)
Giaconi, 2012 [61]	Cross-sectional	584 African American women	Dietary fruits and vegetables Dietary vitamins and carotenoids (vitamins A, C, E, B-complex, α and β carotene, lutein/zeaxanthin, cryptoxanthin, and lycopene	Prevalent POAG, detected by visual field testing and optic disc examination (*n* = 77)	N/A	Retinol (*p*-trend 0.11) Vitamin C (*p*-trend = 0.018) α-carotene (*p*-trend = 0.21) All fruits and fruit juices (*p*-trend = 0.023) Oranges (*p*-trend = 0.002) Peaches (*p*-trend = 0.002) Collard greens/kale (*p*-trend = 0.014)

**Table 2:** Summary of cross-sectional studies of macular pigment optical density (MPOD) and primary open-angle glaucoma.

Author, Year	Participants	MPOD Measurement Technique	Exposure Variable	Mean ± SD (Controls)	Mean ± SD (Cases)	*p*-Value
Igras, 2013 [12]	40 POAG cases 54 healthy controls	HFP	MPOD at 0.5°	0.36 (median)	0.23 (median)	0.03
Siah, 2015 [13]	52 foveal-involved POAG cases 33 controls without foveal involvement	HFP	MPOD at 0.5° ^1^	0.24 ± 0.12	0.15 ± 0.10	<0.001
Ji, 2016 [104]	30 POAG cases 52 healthy controls	Reflectometry	MPOD mean over 7° ^2^	0.14 ± 0.03	0.12 ± 0.03	<0.001
Daga, 2018 [105]	85 POAG cases 22 healthy controls	2-wavelength autofluorescence	MP volume over 7°	7661 ± 3750	8717 ± 3903	0.44

^1^ Significant results also observed for MPOD at 0.25° and 1° targets (*p* < 0.001); ^2^ Significant results also obtained for maximum MPOD (*p* < 0.001).
